# The sweet branch of metabolic engineering: cherry-picking the low-hanging sugary fruits

**DOI:** 10.1186/s12934-015-0389-z

**Published:** 2015-12-09

**Authors:** Rachel Chen

**Affiliations:** School of Chemical and Biomolecular Engineering, Georgia Institute of Technology, 311 Ferst Drive, NW, Atlanta, GA 30332-0100 USA

**Keywords:** Metabolic engineering, Glycoengineering, Glycoproteins, Polysaccharides, Oligosaccharides, Glycoconjugates, Glycans

## Abstract

In the first science review on the then nascent Metabolic Engineering field in 1991, Dr. James E. Bailey described how improving erythropoietin (EPO) glycosylation can be achieved via metabolic engineering of Chinese hamster ovary (CHO) cells. In the intervening decades, metabolic engineering has brought sweet successes in glycoprotein engineering, including antibodies, vaccines, and other human therapeutics. Today, not only eukaryotes (CHO, plant, insect, yeast) are being used for manufacturing protein therapeutics with human-like glycosylation, newly elucidated bacterial glycosylation systems are enthusiastically embraced as potential breakthrough to revolutionize the biopharmaceutical industry. Notwithstanding these excitement in glycoprotein, the sweet metabolic engineering reaches far beyond glycoproteins. Many different types of oligo- and poly-saccharides are synthesized with metabolically engineered cells. For example, several recombinant hyaluronan bioprocesses are now in commercial production, and the titer of 2′-fucosyllactose, the most abundant fucosylated trisaccharide in human milk, reaches over 20 g/L with engineered *E. coli* cells. These successes represent only the first low hanging fruits, which have been appreciated scientifically, medically and fortunately, commercially as well. As one of the four building blocks of life, sugar molecules permeate almost all aspects of life. They are also unique in being intimately associated with all major types of biopolymers (including DNA/RNA, proteins, lipids) meanwhile they stand alone as bioactive polysaccharides, or free soluble oligosaccharides. As such, all sugar moieties in biological components, small or big and free or bound, are important targets for metabolic engineering. Opportunities abound at the interface of glycosciences and metabolic engineering. Continued investment and successes in this branch of metabolic engineering will make vastly diverse sugar-containing molecules (a.k.a. glycoconjugates) available for biomedical applications, sustainable technology development, and as invaluable tools for basic scientific research. This short review focuses on the most recent development in the field, with emphasis on the synthesis technology for glycoprotein, polysaccharide, and oligosaccharide.

## Background

**Fig. 1:**
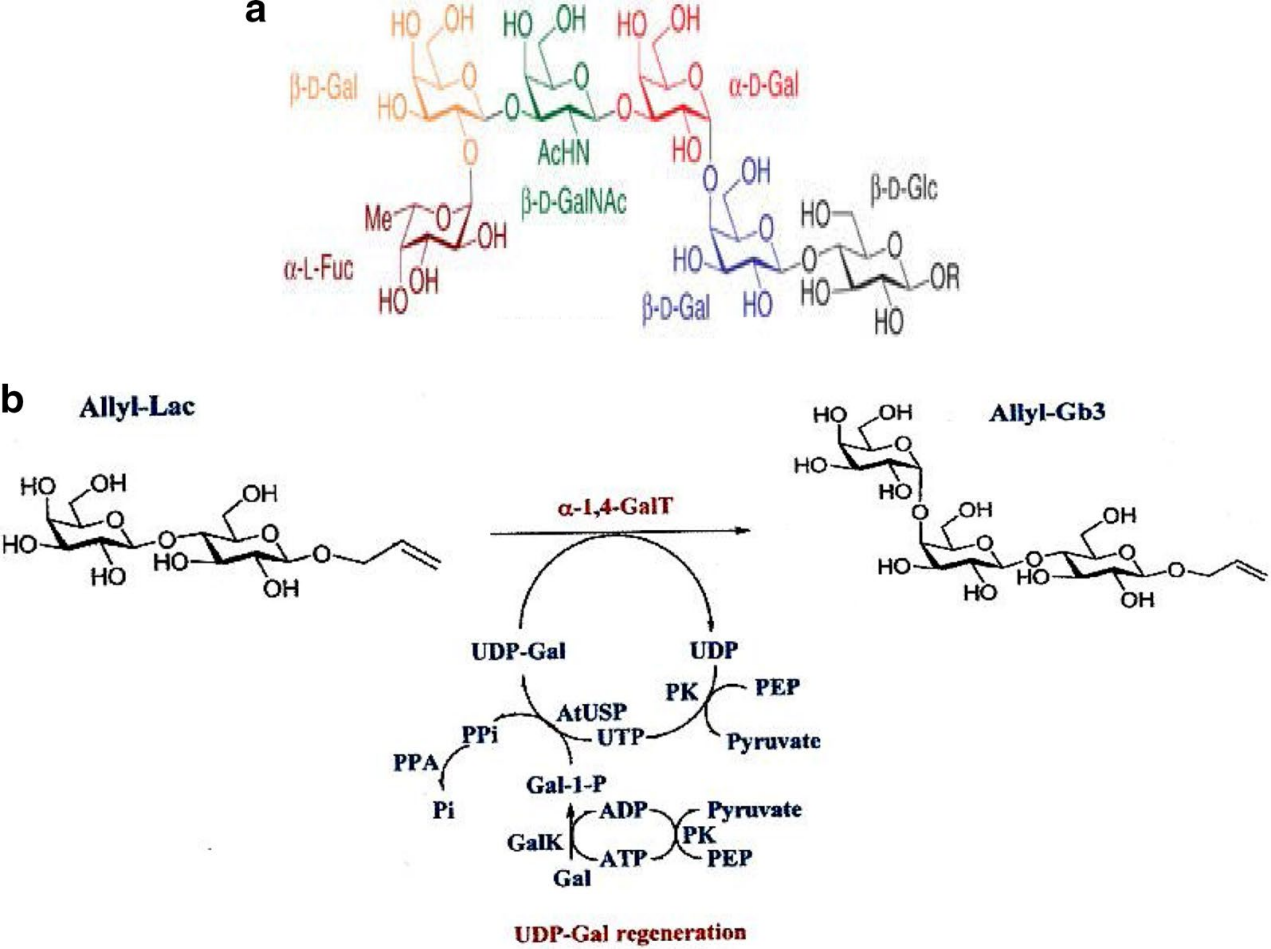
Globo H (**a**) and globo trisaccharide synthesis using in vitro method (**b**) with an α1,4 galactosyltransferase (α 1,4-GalT) and a UDP-Gal regeneration using four enzymes (PK, GalK, AtUSP, PPA) and PEP, ATP, UTP, Gal as starting material. This process leads to one glycosidic bond formation. Abbreviation used: Glc (glucose); Gal (galactose), GalNAc (*N*-acetylgalactosamine), Fuc (fucose); Lac (lactose). Other abbreviations are defined in text or as commonly used in literature (e.g. ATP). Figure 1 is adopted from Tsai et al. (2013) [2]

Glycosylation is the most prevalent and most important co- and post-translational modifications of proteins and lipids. It is estimated that more than 50 % of human proteins are glycosylated. Glycosylation of proteins impacts the processing, distribution, and metabolism as well as the biological functions of most proteins. Glycans on proteins and lipids serve as molecular recognition elements in vital biological processes such as cell growth, differentiation, development, cell–cell interactions, cell migrations, host-microbe interactions, and blood haemostasis [1]. Aberrant glycosylation often signifies a biological transformation of medical significance. An example is a hexasaccharide shown in Fig. 1a, first described as human embryonic intestinal tissue marker and later defined as the SSEA-3 cancer antigen or more commonly known as Globo H antigen, is often associated with tumor aggressiveness [2]. Expression of a specific α-2,6-sialyltransferase was implicated in metastasis from the breast to the brain by increasing cellular adhesion to brain tissue [3]. Altered glycosylation has also been implicated in many different types of diseases and numerous pathological states such as neurodegenerative disorders, rheumatoid arthritis, and cystic fibrosis [4]. Besides present as glycoproteins or glycolipids on cell surfaces, soluble glycans or free oligosaccharides are also important constitutes that play very important roles in biological systems. For example, in human milk, soluble oligosaccharides, with as many as 200 distinct structures, form the third most abundant component in human milk (after lactose and lipid). Increasing evidence suggests that they are anti-infective [5], prebiotic [6], with additional role in modulating immune functions [7]. Additionally, polysaccharides are polymers of sugars, which are synthesized by organisms of all domains and exhibit diverse functions that find numerous in vivo and in vitro applications.

The widespread occurrence of glycosylation and its broad impact in biological processes underscores the importance of studying glycosylation. To study glycans and probe their roles in a biological system significant amount of pure molecules are needed. Besides basic research, there are a wide range of opportunities of utilizing oligosaccharides, polysaccharides, and glycoproteins and other glycoconjugates for diagnosis, vaccine development, as new drug entities, and many other medical applications. Unfortunately, these potential applications are all impeded by the lack of large scale synthesis technology for these molecules. In fact, these molecules are so scarce that it is challenging to gather just enough materials for research, let alone pre- and clinical tests. Take oligosaccharides as an example, very few molecules are available commercially, and for those available for purchase, a typical price is several hundred dollars per milligram, limiting their use to analytical standards. A recent report by National Academy Sciences concludes that glycoscience is critical for advancement of human health and for sustainability of the planet [8]. The report recognizes that the investment in research in this glycoscience has lagged far behind from other aspects of life, especially nucleic acids and proteins [8, 9]. This statement is a true reflect of the current state of glycoscience. Nowadays, a desired DNA sequence can be purchased with very low price (~$0.23 per base) and virtually any protein can be synthesized with recombinant DNA technology. Comparing to the ease of obtaining DNA and proteins, glycans of desired sequence (structure) is very difficult to obtain. Like nucleic acids, proteins and lipids, glycans are one of the four fundamental building blocks of life. The under-development of glycosciences, if not addressed, would ultimately impact our overall understanding of and advances in biological sciences and impede development of medical and other technological fields. Thus there is an urgency to accelerate the research in glycosciences. One critical element in glycoscience research is the availability of glycans or glycoconjugates. Therefore, bringing glycan synthesis technology on the par with those for proteins and DNA/RNA synthesis is urgently needed.

Metabolic engineering, since its inception in late 80s, has grown to be a field impactful in the synthesis of a variety of molecules of commercial and societal importance. Over the past decades, its use in the synthesis of sugar-containing molecules has gained significance, with oligosaccharides, polysaccharides, and glycoproteins particularly noteworthy. In this review, metabolic engineering challenges common to these glycosyltransferase-catalyzed molecules are analyzed and successful examples are showcased to emphasize the power of metabolic engineering as an enabling technology.

### Oligosaccharides

Oligosaccharides are small soluble molecules that serve important roles in biological systems. To obtain significant amount of pure oligosaccharides from natural sources is difficult. This is because they are often heterogeneous, present in minute amount, conjugated to proteins and other biological materials. Chemical synthesis of complex carbohydrates is difficult due to similarity of hydroxyl groups within a sugar molecule and is further complicated by the stereochemistry (a.k.a. anormity) that generates structural isomers when linking two or more monosaccharides together in the synthesis of an oligosaccharide. To differentiate subtle differences between hydroxyl groups requires protection and de-protection groups that add to an already lengthy synthesis route, leading to low yield and often with numerous byproducts requiring complicated workup [10]. Isolating a desired isomer from structurally similar isomers requires additional steps or reagents, increasing complexity and cost of a synthesis process. Biological methods are not as straightforward as proteins since oligosaccharides are not direct gene products. The biogenesis of an oligosaccharide often involves several pathways, further complicated by the regulations inherent in biological processes, such as feedback control and catabolite repression.

Numerous chemical and enzymatic methods were reported for synthesis of oligosaccharides as a mean to provide mg quantities of materials [11–14]. Chemo- and in vitro enzymatic methods are particularly useful to derive non-natural oligosaccharides [15, 16]. The chemo- and enzymatic synthesis method can be illustrated with Globo H (Fig. 1a) as an example. The potential use of this hexasaccharide in anticancer vaccine has motivated chemists to develop several useful synthesis technologies [2]. Most notable is the one-pot programmable method. However, it seems that executing the method still requires highly skilled experts and customized reagents that are only available at limited numbers of elite laboratories. Enzymatic methods offer an alternative, which could be advantageous in reducing steps involved in the synthesis, and byproduct formation [17]. However, in vitro method utilizing isolated glycosyltransferases also present challenges due to the need for activated sugar precursors (or sugar nucleotides) as enzyme substrates. Sugar nucleotides are too expensive to be used in stoichiometric ratio, thus elegant regeneration methods were developed (Fig. 1b). However, these methods still require significant input of expensive high energy compounds in the form of ATP (or GTP, UTP, CTP), phosphoenolpyruvate (PEP) and up to four enzymes in each sugar nucleotide regeneration. In a recent gram-scale synthesis of a trisaccharide intermediate (Globo-trisaccharide, Fig. 1b), 4.55 g PEP was used along with 28 mg ATP and 69 mg UTP, and four recombinant enzymes for the regeneration of UTP-Gal (Fig. 1b). Note that this much material consumption is used to make only one single glycosidic bond, underscoring the difficulty involved in the process. Often biologically significant glycans are complex tetra-, penta- or hexa-saccharides. Thus, their synthesis requires several glycosidic bond formations. While the cost associated with PEP and other high energy compounds may be tolerable for single glycosidic bond formation, further scale up to kg or larger scale, or more complex oligosaccharides using this method would be difficult. Additionally, regeneration of each sugar nucleotide (as shown in Fig. 1b) requires up to four recombinant enzymes, which means four separate fermentation processes for the enzyme production before an in vitro reaction could be set up. Thus, overall, while this method has served well for obtaining limited amount of pure oligosaccharides, further scaling up is problematic due to both cost and steps involved in the synthesis process.

**Fig. 2:**
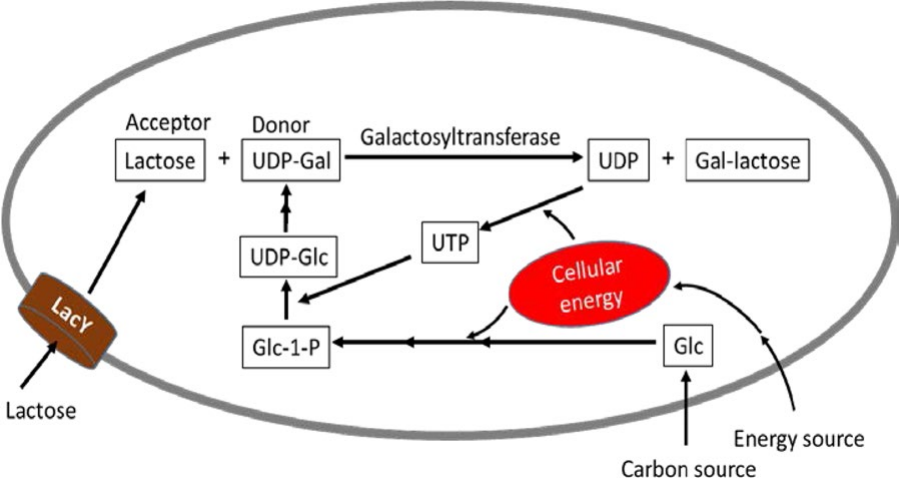
Illustration of a glycosyltransferase catalyzed reaction taking place inside a bacterial cell. Besides an exogenous carbon source in the synthesis of sugar nucleotide, UDP-Gal, an exogenous energy source and its conversion to cellular energy (such as ATP, UTP, etc.) is required

To overcome the aforementioned problems with the in vitro method, in vivo methods such as using bacterial cell coupling was developed in Japan for several simple oligosaccharides [18]. For example, the microbial coupling methods were successfully used to synthesize sialylated lactose to achieve 33 g/L product titer [19]. However, this method is still cumbersome as one single glycosidic bond requires up to four bacterial strains (four fermentation steps). Improvement over this coupling method is the single-strain method developed by Semain et al. [20, 21], in which all enzymes required for sugar nucleotide regeneration and glycosyltransferase enzymes were cloned into a single bacterium. This method has been shown to be successful to generate simple oligosaccharides to titers similar to those obtained with microbial coupling. For example, 25 g/L product titer for 3′-sialyllactose [22] and 34 g/L titer for 6′-sialyllactose [23] were obtained in a high cell density cultivation of engineered *E. coli* using glycerol as carbon and energy source. Potentially, this method has several important advantages over the in vitro enzymatic or microbial coupling methods. The metabolic engineered strains, once developed, could be used in a fermentation process in large bioreactors for kg or larger quantity synthesis. The in vivo method is cost-effective as it does not require any expensive high energy compounds. The valuable oligosaccharides can be produced from inexpensive carbon sources. When *E. coli* is used, cheap medium and fast growth of biocatalyst ensures cost-effective synthesis. Additionally, the synthesis is a single-fermentation process, which could be used to accomplish multiple glycosidic bond formation. Shown in Fig. 2 is an example of a galactosyltransferase enzyme catalyzed reaction with lactose as acceptor sugar and UDP-Gal as donor. At the very minimum, a glycosidic bond formation requires a glycosyltransferase, one sugar acceptor and a donor substrate in the form of sugar nucleotide. Provision of these three essential components is no small feat [10, 24].

#### Glycosyltransferases (GTs)

Since the in vivo one strain approach is dominated by bacteria, such as *Agrobacterium* sp. [25–27], *Corynebacterium glutamicum* [28], *E. coli* [e.g. 29–31]*, and Lactoccous casei* [32], bacterial glycosyltransferases are primarily used as they are more likely to be expressed in these bacterial hosts. Several well-characterized bacterial glycosyltransferases were used in metabolic engineering, including *Helicobacter* fucosyltransferases [33], *Neisseria* and *Photobacterial* sialyltransferases [22, 23], *Neisseria* galactosyltransferases [29], and *Helicobacter* GlcNActransferase [30]. While recent explosive advances in genomics have significantly increased the number of bacterial glycosyltransferases candidates, the increase in new glycosyltransferases used in metabolic engineering has been rather limited. This is because classification of GTs based on sequence similarity or other structure based analysis does not predict their acceptor specificity and the type of linkages [11, 34]. A definitive association of a potential GT gene with its functionality, the substrate specificity, the linkage type requires careful experimental characterizations, which are hampered by the lack of high throughput methods for enzyme assays, and both availability and cost of potential substrates and products. Even with bacterial GTs, an active expression in *E. coli* cannot be assumed a priori. One reason for this is that GTs are usually membrane proteins [35], its active expression and correct folding and insertion into the membrane may require certain factors (such as chaperones) that a host microbe does not have. Additionally, GTs from pathogenic bacteria have certain peculiarity that impacts its heterologous expression. For example, some GTs, such as fucosyltransferases, appear to be dependent on disulfide bond formation for activity [36], and may require host engineering for functional expression in cytosol. Thus, their soluble expression often requires a fusion partner [17, 37], which increases the size of the recombinant protein and the burden on host cells.

#### Acceptor

The challenge of bringing an acceptor sugar into the cytosol lies in the fact that acceptor for the GTF is also a sugar. Additionally, a second sugar is needed as an energy source (Fig. 2). Further, a third sugar (such as fucose and sialic acid) may be needed in the synthesis of donor substrate. A near universal metabolic regulation known as carbon catabolite repression (CCR) prevents cells from engaging more than one sugar at a time. To overcome this challenge, researchers developed some innovative strategies such as producing acceptor in situ [38]. In addition, non-sugar energy source such as glycerol could be used. Furthermore, native monosaccharide and disaccharide transporter could be activated through mutation [21] or inducible overexpression [26]. However, the success of these strategies are highly acceptor specific, no generally applicable method is available to address the issue except passive diffusion, which requires providing an acceptor sugar at a very high concentration. Since acceptor sugars are typically not readily available, large excess of acceptors is generally not feasible in most cases.

#### Donor

Provision of donor substrate requires tapping into host metabolism for necessary energy needed for the synthesis. To provide sufficient sugar nucleotide synthesis, one most commonly used method is selective overexpression of enzymes associated with sugar nucleotide synthesis [31, 32]. This may also include expression of heterologous enzymes and install a completely new pathway. For example, for GDP-fucose synthesis, besides using the *de novo* synthesis, the discovery of a unique bifunctional enzyme FKP from *Bacteroids fragilis* [39] that synthesize GDP-Fuc from fucose, ATP, GTP, provided researchers the salvage pathway for fucosylated oligosaccharides [33, 40]. Another method is to use hosts that have an outstanding capability for sugar nucleotide synthesis. *Agrobacterium* sp. ATCC31749 is a producer of a homo glucose polymer, curdlan. Under optimal conditions, it can accumulate more than 90 g/L curdlan. The strain was engineered to synthesize and regenerate UDP-Gal for production of a disaccharide, LacNAc [25] and a trisaccharide containing α-gal epitope [26]. Multigrams of target oligosaccharides can be derived from 1 liter culture of engineered *Agrobacterium* cells without any input of expensive high energy compounds, demonstrating the effectiveness of this approach. Since polysaccharide synthesis in microbes is common, the strategy of harnessing naturally existing mechanisms to recycle sugar nucleotide for oligosaccharide synthesis could potentially be a more general approach.

In light of the challenges highlighted above, the success of the in vivo one-strain approach is particularly encouraging. The titers of two trisaccharides, 2′-fucosyllactose and 3′-sialyllactose, from high cell density *E. coli* culture could reach 22 and 34 g/L, respectively [23, 33]. However, successful processes with impressive product titers were only reported for relatively small oligosaccharides, mostly trisaccharides. Extending the same strategy for more complex oligosaccharides (containing four or more monosaccharides) resulted in precipitously lower titer, mostly less than 1 g/L [30, 41, 42]. This suggests that the in vivo method cannot be simply extended to complex oligosaccharides. As target oligosaccharides become more complex, the number of glycosidic bond to be formed increases, along with escalated cellular energy demand, and increased number of sugars that a producing strain necessarily have to engage, and more complex of biochemical networks. A satisfactory engineering solution need to confront these challenges before the in vivo one strain approach can be used as a method of choice for complex oligosaccharide synthesis.

### Polysaccharides

Polysaccharides are a class of polymers of diverse chemical structures, physical properties, and biological functions. Over the past decades, numerous polysaccharides have been made into successful commercial products. Xanthan Gum, alginate, bacterial cellulose, curdlan, levan, and hyaluronic acid (a.k.a hyaluronan or HA) are just a few such examples [43]. These polysaccharide products are used in a wide array of applications, including medical treatments, eye surgery, drug delivery, cosmetics, food processing and additives, petroleum recovery. While some of these polysaccharides already capture multi-billion dollar commercial values, they represent only a tip of an iceberg of nature’s bounty. In fact, polysaccharides are produced by organisms of all domains as intracellular, capsular, extracellular products, or as covalently linked components in a large matrix. Microbes and other organisms, however, are evolved to produce the types of polysaccharides with the amounts closely match their own needs. As such, often natural isolates are not optimized to achieve productivity, titer, and yield dictated by a commercially viable bioprocess. Metabolic engineering plays a vital role in strain development for polysaccharide synthesis. This is illustrated by the recent rapid rise of recombinant synthesis technologies for hyaluronan (HA), several of which have successfully moved into the commercial realm [44]. HA is a linear polymer of a repeating disaccharide, β1,3 D-*N*-acetylglucosamin (GlcNAc) β1,4 glucuronic acid (GlcA). It finds application in numerous biomedical procedures such as ophthalmic surgery and osteoarthritis treatment [45]. Traditional methods of production involve extraction from animal tissues and fermentation of pathogenic *Streptococcus*, raising safety concerns. Today, metabolic engineering has made recombinant HA synthesis possible in several GRAS (generally regarded as safe) microorganisms, including *Agrobacterium* sp. [45], *Bacillus subtilis* [46] and *Pichia pastoris* [47], non-pathogenic *E. coli* strains [48, 49], *Corynebacterium glutamicum* [50], food grade strains, *Lactococcus lactis* [51, 52] and *Stretococcus thermophilus* [53]. With process optimization, recombinant strains can approach product titer similar to the *Streptococcus* strain, 5–10 g/L [46, 48]. Much beyond 10 g/L in titer is probably not attainable as the growth medium becomes very viscous, limiting oxygen and other necessary nutrient for the biosynthesis. Recent research has focused on increasing molecular weight of the polymer from recombinant processes, as various applications of HA are highly dependent on the molecular weight of the polymer [54, 55]. While high molecular weight HA is prized for certain medical procedures, the low molecular weight polymer is valuable for cosmetic use. The mechanism governing the molecular weight of HA polymer is yet to be elucidated completely. However, evidence gathered in several producing microbes suggest relative levels of two precursor molecules, UDP-GlcNAc and UDP-GlcA, are one of the important determining factors [56]. Accordingly, the approach taken by Jia et al. by independently controlling the levels of HA synthase and GlcA synthesis was effective in obtaining a high molecular weight HA (up to 5.4 MD) with low polydispersity [57].

HA is only one of glycosaminoglycans (GAGs) of medical importance. Heparin, another important GAG polysaccharide, is used for treatment of coagulation and thrombotic disorders and other medical applications [58]; additionally, chondroitin sulfate (CS) is a GAG used for osteoarthritis treatment. Their traditional association of animal tissues is undesirable. The analogous scenario to HA prompts researchers to exploit metabolic engineering as enabling technology for the production of these complex polysaccharides in non-pathogenic microbes. Could the success of HA be emulated for heparin, CS, and other GAGs? Unlike HA, however, heparin and chondroitin are sulfated GAGs, with additional epimerization along the chain of polymer. Thus the polymer is more complex in chemical composition, compared to HA which is a simple repeats of a disaccharide. *E. coli* K4 and K5 strains produce non-sulfurated polymer backbones that can serve as CS and heparin precursors, respectively. But they lack the sulfotransferases and empimerase needed for the authentic structures. In general, functional expression of human enzymes is challenging in *E. coli*. Thus, the approach worked well so far was a combination of *E. coli* fermentation for precursor production and chemoenzymatic modification in vitro for the final products [58]. Metabolic engineering strategies were used to increase the titer of the precursor molecules. Overexpression of the transcriptional regulator SlyA was effective to raise the titer to 2.6 g/L [59] of K4, a fructosylated chondroitin. Expression of biosynthesis genes of K5 and K4 in laboratory strains is also successful. Optimization of gene expression profiles led to 1.88 g/L haparosan from *E. coli* BL21 strain [60] and 2.4 g/L K4 polysaccharide [61]. To open up opportunity for in vivo modification of the precursor K5, Barreteau and coworkers [62] introduced the K5 synthesis genes to an *E. coli* K12 strain for intracellular production and additionally shown that intracellularly expressed heterologous polysaccharide lyase degraded the polysaccharides into oligosaccharides. Presumably, other modifying enzymes can be introduced and polysaccharides can be modified accordingly. To potentially produce heparin in a one fermentation step, an attempt was also made to engineer CHO cells, which proves to be challenging as while active expression of human *N*-deacetylase/*N*-sulforaansfrase was evident, the increased *N*-sulfo groups were not consistent with pharmacological heparin [63].

Metabolic engineering can also be used for produce non-natural polysaccharides. Yadav and coworkers engineered *Gluconacetobacter xylinus*, the bacterial cellulose producer, to produce a mixed polysaccharide with glucose and GlcNAc as monomers [64]. The copolymer is more readily biodegradable than cellulose. The success of this approach relies on the promiscuous nature of the cellulose synthase. In the future, protein engineering can be used, in combination of metabolic engineering, to tailor properties of a polysaccharide for a particular application. For example, fucosylated chondroitin sulfate from sea cucumber was recently shown in an animal model to activate insulin signaling [65]. If K4 polymerase is promiscuous enough or an engineered version could be found to incorporate fucose into K4, it is conceivable that a new type of polymer, fucosylated K4, could be produced from *E. coli*.

While above examples are skewed toward medically important polysaccharides, this certainly does not reflect the diverse polysaccharides produced by various organisms and their potential applications. Polysaccharides are also produced naturally by soil microbes in vital processes such as symbiosis. One such polysaccharide is succinoglycan produced by *Sinorhizobium meliloti*. A recent work demonstrated that enhanced production of succinoglycan by overexpression *exoY* gene promoted the symbiosis with the host plant *Medicago truncatula* [66]. This is metabolic engineering in a non-traditional sense, as the product of metabolic engineering is not isolated from a bioreactor; rather the product is used in situ in a process. In the future, this may also become an important use of metabolic engineering. Scleroglucan is another example of polysaccharides useful outside medical field. Its non-medical application ranges from oil recovery, food processing, and cosmetics. The recently sequenced genome of *Sclerotium rolfsii*, opens up metabolic engineering opportunities to produce the fascinating molecule in non-pathogenic microbes [67].

### Glycoproteins

Biopharmaceutical industry has grown to be an industry with hundreds of products and market value over $100 billion [68, 69]. About 70 % of the human protein therapeutics are glycoproteins [70]. Not surprisingly, since glycoforms are crucial for the functionality of the protein and therapeutic outcomes, mammalian cell lines, most prominently CHO, are used for glycoproteins destined for human use. Even with CHO cells, however, the glycosylation patterns are not human enough. One difference between human and CHO protein is the linkage of sialic acid. Human sialylated proteins have both α2,6 and α2,3 linkages, whereas CHO only produces α2,3-linked proteins. This can be addressed, as Bailey described in a 1991 review, by metabolically engineering, specifically introducing α2,6-sialyltransferase into CHO [71]. Two recent reports indicate this strategy is successful in increasing sialylation of commercially important glycoproteins, immunoglobulin (IgG) [72] and erythropoietin (EPO) [73]. In the case of IgG, introducing α2,6-sialyltransferase is sufficient to generate protein with this new linkages, increasing the total sialic acid content of the recombinant proteins up to two fold [72]. In the case of EPO, the goal was to increase sialylation of highly branched glycans, since they are more important for therapeutic functions. Thus, besides expression of α2,6-sialyltransferase, two additional glycosyltransferases were introduced to CHO cells, successfully increasing the tri- and tetra-sialylated structures. As a result, these structures account for 90 % of the total sialic acid of EPO produced by metabolically engineered CHO [73].

Besides increasing sialylation and making human-like glycoforms, metabolic engineering could be used to simplify or install new glycoforms on a recombinant protein. For example, glycoforms can be simplified to assist structure studies and enhance homogeneity. Sealover and coworkers used zinc-finger nuclease (ZFN) genome editing tools to knockout the gene encoding an essential branching enzyme, the mannosyl (α1,3)-glycoprotein-β1,2-acetylglcosaminyltransferase (MGAT1), creating neoglycoproteins with Man_5_GlcNAc_2_ structures, useful for x-ray crystallography studies and for vaccines targeting mannose receptors [74]. To install a single trisaccharide glycan on recombinant protein, the Man_5_GlcNAc_2_ structure was further trimmed down to a single GlcNAc by expressing a fungal endo-β-*N*-acetylglucosaminidase targeted to Golgi. The single sugar glycan (GlcNAc) was subsequently extended to a trisaccharide by addition of a galactose and a sialic acid by coexpression of two respective glycosyltransferases [75]. This metabolic engineering approach has the advantage to drastically reduce the complexity of glycan structures on a glycoprotein thereby improving glycan homogeneity. The Zinc Finger nuclease gene editing technique was also used to modify *O*-glycosylation of human cell lines by knocking out chaperones in the folding of key glycosyltransferases, resulting truncated glycans, which were enriched by affinity chromatography for further analysis [76]. Newer and more powerful genome editing techniques such as CRISPR/Cas9 system is expected to make editing of mammalian cell lines easier and less prone to artifacts.

Among all alternatives to mammalian cell culture, the methylotrophic yeast *Pichia pastoris* based technology seems to be most advanced. Both* N*- and* O*-glycoproteins resembling human glycoforms could be produced from *P. pastoris* [77]. Recent research significantly improved site occupancy to almost 100 % [78, 79]. Process development and optimization, and scale up studies have been carried out up to several thousand liters. Product titers reached a g per liter level and importantly, the engineered yeast strain appeared to be robust, and glycosylation patterns are consistent across several orders of magnitude change in cultivation volumes [80]. A comparison in glycosylation of EPO produced by *Pichia* and CHO showed that EPO produced by *Pichia* is more homogenous with bi-antennary whereas CHO produced EPO contained additional tri- and tetra-antennary structures [81], known to be more active for its therapeutic function, suggesting that the state of art yeast glycoprotein technology leaves room for improvement and for alternative technologies. Indeed, efforts in engineering plant and insect cells offer increasingly realistic prospect of using these potentially more cost-effective hosts for glycoprotein production. Researchers recently achieved a significant metabolic engineering milestone in plant, making plant cells perform mammalian-like glycosylation of human EPO with tri- and tetra-sialylation. This is significant as this is the only system other than CHO, capable of this type of glycosylation [82]. A major goal in engineering insect cells is to elongate the insect glycan to human glycans which typically have penultimate galactose and terminal sialic acid. Recent efforts focused on making cells to perform human like glycosylation by engineering glycosyltransferase targeting [83] and inducible coordinated expression of glycosylation [84], and reduce immunogenic fucosylation [85].

Ever since the first functional transfer of bacterial *N*-glycosylation in *Campylobacter jejuni* into *E. coli* [86], the prospect of making glycoproteins in *E. coli* has generated much excitement. The progress over the last decades indeed has made the bacterial glycosylation much closer to be exploited as a realistic technology [70, 87]. Two areas of applications are particularly promising. The first is to produce glycoprotein vaccine with carbohydrate antigen presented on the surface of a protein carrier. A recent paper on the production of a recombinant vaccine against *Burkhoderia pseudomallei* illustrates how a protein bearing specific polysaccharide antigen can be synthesized in *E. coli* [88]. The polysaccharide antigen in question requires 15 genes, which was introduced in *E. coli* via a plasmid. *E. coli* strain was also modified by deletion of *waaL* and *wecA*, encoding enzymes that either prevent or interfere with the glycan ligation to AcrA, a *Campylobacter* glycoprotein. The ligation is catalyzed by PglB, the *Campylobacter jejuni* oligosaccaryltransferase [88]. Similar approach was used to develop multivalence vaccine when the carrier protein was an antigen itself and was conjugated with one or more antigenic glycans. Wacker and coworkers demonstrated a trivalent vaccine against *Staphylococcus aureus* [89]. Besides bacterial antigens, other glycan such as human Lewis antigen associated with autoimmune diseases could be presented on a protein using bacterial glycosylation systems [90], thanks to the relaxed glycan substrate specificity of PglB. Although the first glycoprotein bearing Lewis antigen used a combination of in vivo and in vitro methods due to lack of GDP-fucose biosynthesis capability of *E. coli* native strains, this deficiency can be addressed using metabolic engineering strategies. Thus we could expect that in the future a designer glycoprotein vaccine carrying one or more antigens from diverse biological sources could be made by metabolically engineered *E. coli* cells in a one fermentation step.

Recombinant bacterial glycosylation systems can also be exploited for production of glycoprotein therapeutics currently on market, or hundreds of others in various stages of development. While the mechanism and methodology are the same as those used for vaccine development, the current research in this applications focuses on increasing glycoprotein titers, glycosylation efficiency (occupancy), and authenticity of glycans, as most of these therapeutic proteins are for human uses [91–93]. The bacterial system, while having the potential to revolutionize the manufacture technology, currently is not competitive with the mammalian technology, nor with any of the alternatives (*Pichia*, plant, and insect cells) reviewed above. The titer of glycoproteins from *E. coli* cells is in mg/L range, 2–3 orders of magnitude lower than those of other technologies. Glycan structures on glycoproteins derived from bacterial systems are still quite a distance from those of human or even mammalian like. For example, no sialylated glycoproteins has made from bacterial system. Glycosylation efficiency is also mostly lower than <50 %, sometime 1 %, far lower than desired complete glycosylation [94]. However, these challenges are not insurmountable, especially considering the relatively short period of time the progress has made. Recent successes in expressing human sialyltranferase (ST6) [95] and human glycosyltransferase GalNAc T2 [96] in *E. coli* bring it closer to a bacterial technology capable of producing humanized glycoproteins.

## Conclusions

Glycosidic bond formation catalyzed by glycosyltransferase enzymes is in the center of the synthesis of most glycan structures in nature. Oligosaccharides, polysaccharides, and glycoproteins share the commonality that requires glycosyltransferases in their synthesis, differing only in the nature of the acceptors. Not reviewed above, glycolipids are similarly synthesized with a lipid as an acceptor. Glycosylated natural products, predominantly represented by some antibiotics such as vancomycin, also requires glycosyltransferase enzyme in the synthesis. Therefore, from a metabolic engineering point of view, they share much of the synthesis challenges. These include, as analyzed above, the high energy demand due to the need for sugar nucleotides as precursors, the complexity of metabolic pathways and regulations involved, and the adequate supply of acceptors when and where the glycosyltransferases are most active. Represented by 2′-fucosyllactose, HA, and EPO, the success in bringing highly valuable oligo- and poly-saccharides, and glycoproteins to commercial production demonstrates the power of this branch of metabolic engineering. On the other hand, given the enormous diversity and significant complexity of saccharide-containing structures, a handful of molecules attaining commercial success can only qualify as a promising beginning. In fact, the surface of the gigantic glyco-sphere has barely scratched. Providing scientists with hundreds and thousands of glycans in quantities sufficient to probe their structure and function relationships and supplying clinicians with selective compounds (such as Globo H and heparin in kg quantities) for clinical studies in a cost effective manner are challenges before metabolic engineers and synthetic biologists. The inherent challenges in complex carbohydrates demand innovative metabolic engineering strategies beyond a simple extension of those used in successful examples reviewed here. Thus, this branch of metabolic engineering will sure grow and the rewards in this interdisciplinary field are sweet and plentiful.
